# One-year patient outcomes based on lung morphology in acute respiratory distress syndrome: secondary analysis of LIVE trial

**DOI:** 10.1186/s13054-022-04036-7

**Published:** 2022-06-04

**Authors:** Florian Blanchard, Thomas Godet, Stephanie Pons, Natacha Kapandji, Matthieu Jabaudon, Vincent Degos, Lucile Borao, Adrien Bougle, Antoine Monsel, Emmanuel Futier, Jean-Michel Constantin, Arthur James

**Affiliations:** 1 GRC 29, AP-HP, DMU DREAM, Department of Anesthesiology and Critical Care, Pitié-Salpêtrière Hospital, Sorbonne University, Paris, France; 2 Department of Perioperative Medicine, CHU Clermont-Ferrand, Clermont-Ferrand, France; 3 GReD, CNRS, INSERM, Université Clermont Auvergne, Clermont-Ferrand, France; 4 Réanimation Chirurgicale Polyvalente, GH Pitié-Salpêtrière, 47-83 Boulevard de l’Hôpital, 75013 Paris, France

**Keywords:** ARDS, Critical illness, Follow-up study, ICU, Long-term outcomes, Phenotype, Quality of life

## Abstract

**Background:**

Acute respiratory distress syndrome (ARDS) has different phenotypes and distinct short-term outcomes. Patients with non-focal ARDS have a higher short-term mortality than focal ones. The aim of this study was to assess the impact of the morphological phenotypes of ARDS on long-term outcomes.

**Methods:**

This was a secondary analysis of the LIVE study, a prospective, randomised control trial, assessing the usefulness of a personalised ventilator setting according to lung morphology in moderate-to-severe ARDS. ARDS was classified as focal (consolidations only in the infero-posterior part of the lungs) or non-focal. Outcomes were assessed using mortality and functional scores for quality of life at the 1-year follow-up.

**Results:**

A total of 124 focal ARDS and 236 non-focal ARDS cases were included. The 1-year mortality was higher for non-focal ARDS than for focal ARDS (37% vs. 24%, *p* = 0.012). Non-focal ARDS (hazard ratio, 3.44; 95% confidence interval, 1.80–6.59; *p* < 0.001), age, McCabe score, haematological cancers, SAPS II, and renal replacement therapy were independently associated with 1-year mortality. This difference was driven by mortality during the first 90 days (28 vs. 16%, *p* = 0.010) but not between 90 days and 1 year (7 vs. 6%, *p* = 0.591), at which point only the McCabe score was independently associated with mortality. Morphological phenotypes had no impact on patient-reported outcomes.

**Conclusion:**

Lung morphologies reflect the acute phase of ARDS and its short-term impact but not long-term outcomes, which seem only influenced by comorbidities.

*Trial registration*: NCT 02149589; May 29, 2014.

**Supplementary Information:**

The online version contains supplementary material available at 10.1186/s13054-022-04036-7.

## Background

Since its first description up to its association with COVID-19, acute respiratory distress syndrome (ARDS) continues to represent a challenging feature in the intensive care unit (ICU) [1]. Mortality due to ARDS remains high without any decrease since the late 1990s [2]. Multiple therapeutics have failed to enhance survival, and quality of life is highly impacted in ARDS survivors [3]. The lack of promising interventions calls for a better targeting of interventions to a subset of patients [4]. Thus, ARDS research has moved from a simple syndromic clinical description to a more complex and broader field of subphenotypes [5].

Various ARDS phenotypes have been described over the past 10 years based on a range of pulmonary physiologic abnormalities [5]. Important investigations focusing on inflammatory biomarkers have been reported using latent class analysis [6]. Hyper- and hypo-inflammatory phenotypes have been described with worse outcomes for the hyper-inflammatory phenotype. Furthermore, post hoc analysis of negative randomised clinical trials found improved outcomes in the treatment arm for patients with hyper-inflammatory phenotype [7]. Similarly, analysis of lung morphology identified two subgroups—focal and non-focal ARDS—with major differences in lung physiology. Non-focal ARDS is associated with worse respiratory mechanics, and morphological phenotypes may influence the response to positive end-expiratory pressure, recruitment manoeuvres, and prone position [8, 9]. Recently, the LIVE study assessed the use of personalised ventilation based on lung morphology [10]. Although the trial was negative, post-hoc analysis suggested promising results for better personalised mechanical ventilation based on lung morphology.

Aside from important in-hospital mortality rates, 1-year mortality is high, and ARDS-related markers of severity are not associated with mortality among ARDS survivors. However, comorbidities and not living at home prior to admission were reported as independent predictors of 1-year mortality [11]. A recent study by Hashem and colleagues assessing long-term outcomes in inflammatory subphenotypes found that those phenotypes largely reflect the acute phase of illness and its short-term impact, with little to no impact on survival beyond 90 days [12]. Although the association between non-focal ARDS and higher 90-day mortality was previously reported, longer-term survival and quality of life have never been investigated in focal and non-focal ARDS. Therefore, our main objective was to assess the long-term outcomes (1 year) of focal and non-focal ARDS. Our secondary objective was to assess the impact of personalised ventilation protocol using a secondary analysis of the prospective LIVE trial after a 1-year follow-up.

## Methods

### Design and patients

We conducted a secondary preplanned analysis on the long-term outcomes of patients enrolled in the LIVE study, as described in the original protocol [13]. The LIVE study was a prospective, multicentre, stratified, parallel-group, single-blind randomised controlled trial in 20 university and non-university French ICUs [10]. Patients with moderate-to-severe ARDS, according to the Berlin definition, were characterised by local site investigators as having focal ARDS (presence of consolidations localised only in the lower and back parts of the lungs) or non-focal ARDS [14]. Lung morphology was assessed before randomisation using a CT scan of the whole lung or chest X-ray when the severity of the patient was not compatible with transport. Patients were randomly assigned to a ventilation protocol adjusted on the basis of lung morphology (personalised group) or to a standard strategy in line with traditional care (control group). The detailed protocol is available in the online supplement.

In the LIVE trial, 24% of patients were misclassified as focal or non-focal ARDS before randomisation (see LIVE trial for more explanation [10]). We performed an intention-to-treat analysis that included all participants who were randomly assigned to treatment, except those who withdrew consent and those who were found to be ineligible because they met the exclusion criteria. Next, we performed a per-protocol analysis in which misclassified patients in the personalised group were excluded due to a breakdown in the ventilation protocol. Misclassified patients in the control group were not excluded because they were not misaligned with ventilator strategy, which, by definition, is not related to lung morphology.

The study took place in two steps. First, long-term outcomes were assessed based on lung morphology phenotypes: focal versus non-focal ARDS. Then, the impact of a personalised ventilation protocol (i.e. personalised vs. control group) was assessed on the same outcomes. Only the per-protocol analysis was used to compare focal and non-focal ARDS to avoid any bias induced by misclassified patients. However, both the intention-to-treat and the per-protocol analyses were used to compare the personalised and control groups. The results of the personalised and control groups are reported in the Supplemental Data available online.

### Ethics

According to current French law, the original trial was approved by the “Institutional Review Board of Clermont-Ferrand, France” (ID RCB 2013-A01756-39) and registered on *ClinicalTrials.gov* (NCT 02149589) [15]. Before any inclusion, written informed consent was obtained from patients or their relatives. They could refuse to participate at any time, and their decisions were recorded in patient files.

### Outcomes

Patient’s survival was assessed, after a 1-year follow-up, between focal and non-focal ARDS and between personalised and control groups. Early mortality was defined as mortality in the first 90 days. Late mortality was defined as mortality between day 90 and 1 year. Further, at the 1-year follow-up, survivors were evaluated by trained research staff who were blinded to treatment allocation. Patient-reported outcome measurements included age- and sex-adjusted physical function and mental health domain scores of the Medical Outcomes Study Short Form 36 (SF-36) instrument [16]; anxiety and depression symptoms from the Hospital Anxiety and Depression Scale (HAD) subscale scores [17]; and effects of fatigue on quality of life from the Modified Fatigue Impact Scale (MFIS) [18]. SF-36 ranges from 0 to 100 with 8 categories and sums up into the physical component summary and the mental component summary. HAD score is divided into anxiety and depression subscales, both ranging from 0 to 21. The presence of anxiety or depression was defined as a subscale over 7 [19]. MFIS total score ranges from 0 to 84 with three subscales: physical (range 0–36), cognitive (range 0–40), and psychosocial (range 0–8). Based on previous studies, patients with an MFIS of over 38 were described as having fatigue [20].

### Statistical analysis

Given that this was a secondary analysis of the LIVE study, no sample size calculation was required a priori. A descriptive analysis was performed on all patients and survivors. Quantitative variables are expressed as mean (standard derivation [SD]) or median (interquartile range [IQR], 25–75%) and compared using Student’s t test or the Mann–Whitney U test, as appropriate. Categorical variables are expressed as numbers (%) and compared using the chi-square test or the Fisher’s exact test as appropriate.

Survival curves were plotted using the Kaplan–Meyer method, and comparisons were made using the log-rank test and a Cox model. To assess the impact of lung morphology phenotypes (focal vs. non-focal ARDS) on mortality, significant variables during the univariable analysis (threshold of *p* < 0.10) or any variable known to be associated with mortality during ARDS were included in a Cox model. Multivariable analysis was performed on complete cases. A sensitivity analysis with multiple imputations was conducted to deal with missing data. Additional information about the process use for multiple imputations is reported in Additional file 1.

Statistical analyses used to assess the impact of a personalised ventilation protocol adjusted on the basis of lung morphology (personalised vs. control group) are reported in Additional file 1.

To compare the functional outcomes of the SF-36 instrument with previous reports [3, 21, 22], *z*-scores were created by standardising the values (mean = 50, standard deviation = 10; range 0–100, with a higher score indicating better function) using previously described methods [23] and a French cohort of healthy patients [24]. A Mann–Whitney U test was used to assess the difference between the z-scores and 50. Because 35% of patients missed the functional outcomes assessment, a sensitivity analysis with imputation was conducted to deal with missing data. Additional information about the process use for imputation is reported in Additional file 1. A *P* value of ≤ 0.05 was considered to be statistically significant. Statistical analyses were carried out using R version 4.1.2 for macOS® (https://www.r-project.org, accessed November 2021).

## Results

### Population

**Fig. 1:**
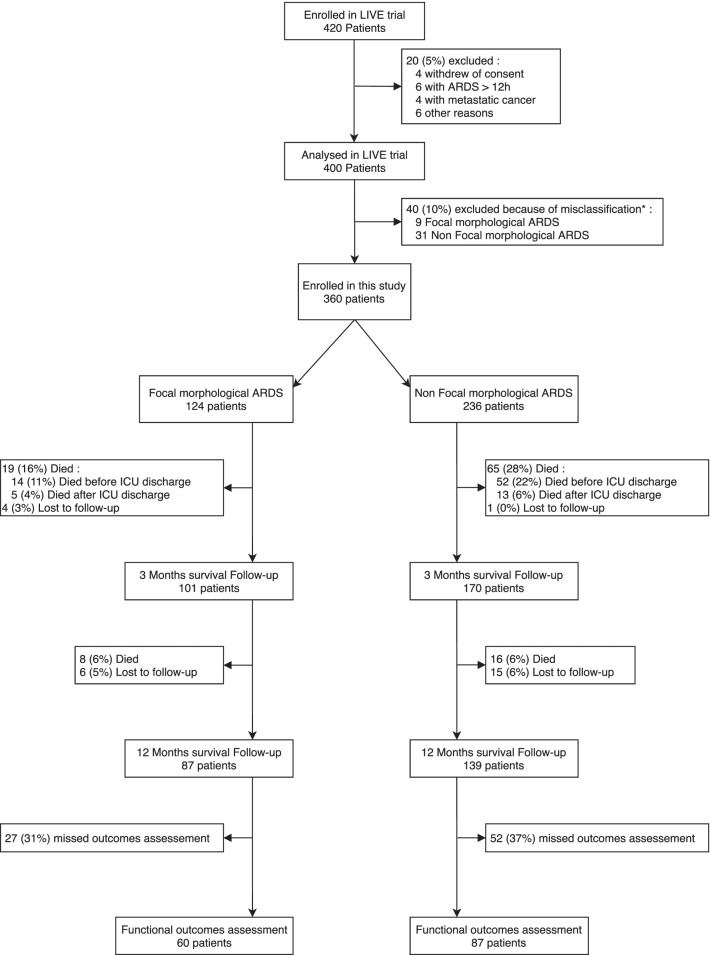
Flow chart: Shown is the recruitment of the cohort for analysis comparing focal and non-focal ARDS. *Misclassified patients (focal ARDS characterised as non-focal ARDS or non-focal ARDS characterised as focal ARDS) in the intervention arm of the LIVE study (patients with a personalised ventilation protocol based on the morphological phenotypes) were excluded due to a breakdown in the ventilation protocol. ARDS: Acute respiratory distress syndrome

From June 2014 to February 2017, 420 patients were included in the LIVE study. Flow charts of the study are reported in Fig. 1 and Additional file 1: Fig. S1. Sixty patients were excluded from the per-protocol analysis, as described in Additional file 1: Fig. S1. Of the remaining 360 patients, 124 (34%) and 236 (66%) had focal and non-focal ARDS, respectively (Fig. 1). Information for survival at 1 year was available for 334 patients (93%) (114 and 220 patients with focal and non-focal ARDS, respectively). Data on the population included to compare the personalised and control groups are reported in the online supplement.

### Mortality according to lung morphology

Patient baseline data for the per-protocol cohort and for the focal and non-focal ADRS groups are summarised in Table 1. There were more males, and BMI was higher in the focal ARDS group. Plateau pressure, PEEP, and driving pressure were higher in non-focal ARDS. Ventilator-free days to day 28 and ICU length of stay were not different between the groups.

**Table 1:** Characteristic and clinical baseline patient data

Variables	Both groups* n = 360	Focal ARDS *n* = 124	Non-focal ARDS *n* = 236	*P* value^‡^
*Baseline patient data*				
Age, years, mean [SD]	61 [15]	61 [15]	62 [15]	0.301
Males, *n* (%)	266 (74%)	101 (81%)	165 (70%)	**0.018**
Female, *n* (%)	94 (26%)	23 (19%)	71 (30%)	**0.018**
COPD, *n* (%)	32 (9%)	16 (13%)	16 (7%)	0.052
Chronic renal failure, *n* (%)	6 (2%)	1 (1%)	5 (2%)	0.355
Non-haematological cancers, *n* (%)	41 (11%)	13 (10%)	28 (12%)	0.695
Haematological cancers, *n* (%)	14 (4%)	3 (2%)	11 (5%)	0.296
Diabetes, *n* (%)	41 (11%)	14 (11%)	27 (11%)	0.966
Arterial hypertension, *n* (%)	75 (21%)	26 (21%)	49 (21%)	0.964
Smoking, *n* (%)	10 (3%)	3 (2%)	7 (3%)	0.764
Alcohol disturbance, *n* (%)	46 (13%)	13 (10%)	33 (14%)	0.345
Vasculopathy, *n* (%)	66 (18%)	23 (19%)	43 (18%)	0.939
Cardiopathy, *n* (%)	31 (9%)	12 (10%)	19 (8%)	0.601
BMI, kg/m^2^, mean [SD]	26.3 [5.1]	27.1 [5.0]	25.9 [5.1]	**0.030**
McCabe score				0.924
0, *n* (%)	249 (69%)	85 (69%)	164 (69%)	
1, *n* (%)	98 (27%)	35 (28%)	63 (27%)	
2, *n* (%)	13 (4%)	4 (3%)	9 (4%)	
*Baseline intensive care data*				
Medical condition at admission, n (%)	300 (83%)	99 (80%)	201 (85%)	0.197
SAPS II, mean [SD]	51 [16]	52 [16]	51 [16]	0.629
SOFA, mean [SD]	9 [4]	10 [4]	9 [4]	0.182
PaO_2_/FiO_2_, mean [SD]	117 [42]	115 [40]	118 [43]	0.704
Plateau pressure, cm H_2_O, mean [SD]^**†**^	24 [6]	23 [4]	24 [6]	**0.029**
PEEP, cm H_2_O, mean [SD]^**†**^	10 [3]	9 [3]	10 [4]	**0.047**
Driving pressure, cm H_2_O, mean [SD]^**†**^	14 [5]	13 [4]	14 [5]	**0.038**
Tidal volume over ideal body weight, mL/kg, mean [SD]^**†**^	6.6 [1.1]	6.6 [1.1]	6.5 [1.1]	0.218
FiO_2_, %, mean [SD]^**†**^	77 [20]	75 [20]	77 [21]	0.361
Steroids, *n* (%)	80 (22%)	22 (18%)	58 (25%)	0.177
Shock at baseline, *n* (%)	222 (62%)	81 (65%)	141 (60%)	0.301
Renal replacement therapy, *n* (%)	91 (25%)	34 (27%)	57 (24%)	0.582
Primary lung injury				0.341
Pneumonia, *n* (%)	174 (48%)	52 (42%)	122 (52%)	
Non-pulmonary infection, *n* (%)	73 (20%)	28 (23%)	45 (19%)	
Aspiration, *n* (%)	79 (22%)	31 (25%)	48 (20%)	
Trauma, *n* (%)	7 (2%)	4 (3%)	3 (1%)	
Other, *n* (%)	27 (8%)	9 (7%)	18 (8%)	
*Outcomes*				
Ventilator-free days to day 28, days, mean [SD]	13 [10]	15 [9]	13 [10]	0.151
ICU LOS, days, mean [SD]	17 [16]	17 [14]	17 [17]	0.917

ARDS, acute respiratory distress syndrome; BMI, body mass index; COPD, chronic obstructive pulmonary disease; FiO_2_, fraction of inspired oxygen; ICU, intensive care unit; LOS, length of stay; PaO_2_, partial pressure of oxygen in arterial blood; PEEP, positive end expiratory pressure; SAPS II, simplified acute physiology score II; SOFA, Sequential Organ Failure Assessment score

*Both groups reported patients in the per-protocol analysis

^‡^p values are reported between focal and non-focal ARDS (*p* value < 0.05 are in bold type)

^†^The reported variables were assessed during the first day after inclusion

**Fig. 2:**
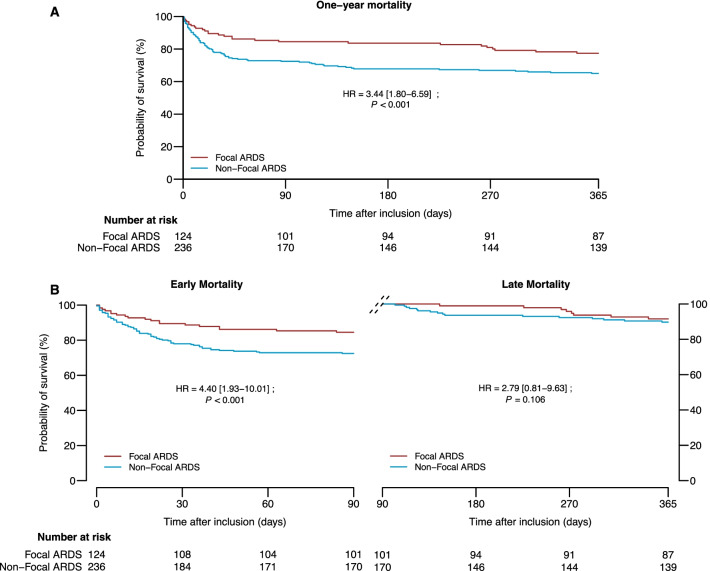
Mortality assessment during a 1-year follow-up between focal and non-focal ARDS. **A** Survival analyses in the per-protocol cohort by morphological phenotypes during a 1-year follow-up (log-rank test: *p* = 0.012, HR = 3.44 [1.80–6.59] *p* < 0.001, with higher mortality among non-focal ARDS), **B** Early and late mortality defined as mortality during the first 90 days and between day 90 and 1 year, respectively. One-year mortality was driven by early mortality (log-rank test: *p* = 0.010, HR = 4.40 [1.93–10.01] *p* < 0.001) but not by late mortality (log-rank test: *p* = 0.591, HR = 2.79 [0.81–9.63] *p* = 0.106). Survival curves were realised using the Kaplan–Meyer methods. ARDS: acute respiratory distress syndrome

The 1-year mortality was higher in non-focal ARDS patients compared with focal ARDS patients (81 [37%] vs. 27 [24%], respectively; log-rank test: *p* = 0.012) (Fig. 2A). In the multivariate analysis, non-focal ARDS (hazard ratio (HR), 3.44; 95% confidence interval (95%CI), 1.80–6.59; *p* < 0.001), age (HR, 1.04; 95%CI, 1.02–1.06; *p* < 0.001), McCabe score (HR, 1.51; 95%CI, [1.04–2.19]; *p* = 0.029), haematological cancers (HR, 2.24; 95%CI, 1.02–4.97; *p* = 0.045), SAPS II (HR, 1.02; 95%CI, 1.00–1.04; *p* = 0.047), and renal replacement therapy (HR, 1.71; 95%CI, 1.04–2.79; *p* = 0.033) were independently associated with 1-year mortality (Additional file 1: Table S1).

The difference in 1-year mortality was driven by early mortality (65 non-focal ARDS patients [28%] vs. 19 focal ARDS patients [16%]; log-rank test: *p* = 0.010) but not by late mortality (16 non-focal ARDS patients [7%] vs. 8 focal ARDS patients [6%]; log-rank test: *p* = 0.591) (Fig. 2B). Non-focal ARDS, age, SAPS II, and renal replacement therapy were independent predictors of early mortality (Table 2). Only the McCabe score was associated with late mortality (Table 2). During the first 90 days, death was directly related to ARDS in 35% and 37% of patients with focal and non-focal ARDS, respectively (*p* = 0.89). Underlying disease was accountable for 65% and 63% of deaths in focal and non-focal ARDS, respectively.

**Table 2:** Cox model result of the multivariate analysis for early and late mortality

Predictors	Early mortality		Late mortality	
	Hazard ratio*	*p* value	Hazard ratio*	*p* value
Non-focal ARDS	4.40 [1.93–10.01]	** < 0.001**	2.79 [0.81–9.63]	0.106
Age	1.04 [1.02–1.07]	** < 0.001**	1.03 [0.99–1.07]	0.126
Males	0.85 [0.45–1.60]	0.618	0.82 [0.25–2.74]	0.751
Haematological cancers	2.27 [0.95–5.43]	0.064	1.73 [0.17–17.70]	0.642
Non-haematological cancers	2.09 [0.95–4.57]	0.066	1.48 [0.39–5.68]	0.566
COPD	1.33 [0.55–3.25]	0.525	2.30 [0.54–9.84]	0.263
McCabe	1.11 [0.71–1.73]	0.645	3.56 [1.45–8.72]	**0.005**
BMI	0.99 [0.94–1.05]	0.789	0.93 [0.84–1.04]	0.213
SAPS II (without age)^†^	1.02 [1.00–1.04]	**0.047**	1.02 [0.98–1.07]	0.357
PaO_2_/FiO_2_	1.00 [1.00–1.01]	0.586	0.99 [0.98–1.00]	0.065
Plateau pressure	1.12 [0.91–1.37]	0.285	0.86 [0.59–1.25]	0.426
PEEP	0.90 [0.74–1.10]	0.305	1.07 [0.72–1.58]	0.740
Driving pressure	0.91 [0.74–1.11]	0.360	1.16 [0.80–1.68]	0.426
Shock	1.24 [0.67–2.27]	0.493	1.55 [0.48–5.06]	0.466
Mechanical ventilation over 48 h	2.08 [0.61–7.09]	0.243	0.71 [0.15–3.28]	0.659
Steroid	1.13 [0.63–2.03]	0.689	1.66 [0.54–5.12]	0.381
Renal replacement therapy	2.42 [1.40–4.20]	**0.002**	0.29 [0.06–1.46]	0.133
Intervention arm in LIVE study^$^	0.65 [0.38–1.11]	0.115	1.66 [0.56–4.96]	0.362

Early and late mortality were defined as mortality in the first 90 days and mortality between day 90 and 1 year, respectively (p values < 0.05 are in bold type)

ARDS, acute respiratory distress syndrome; FiO_2_, fraction of inspired oxygen; CI95%, 95% confidence interval; PaO_2_, partial pressure of oxygen in arterial blood; SAPS II, Simplified Acute Physiology Score II

*Results are reported as hazard ratio [CI95%]

^†^SAPS II predictors include SAPS II without age due to the presence of the age already in the model

^$^Intervention arm is explained in the LIVE study report

There were no missing data for the included variables, except for plateau pressure, PEEP, and driving pressure, which were unavailable for 83 (23%), 14 (4%), and 88 (24%) patients, respectively. A sensibility analysis with multiple imputations for missing data confirmed the previous multivariate analysis. (Additional file 1: Table S2).

### Quality of life according to lung morphology

A total of 226 patients survived after a 1-year follow-up and were eligible for functional outcomes assessment. We noted that 79 patients (35%) missed the functional outcomes assessment (27 [31%] and 52 [37%] patients with focal and non-focal ARDS, respectively) (Fig. 1). There was no difference between patients who did and did not complete the functional outcomes assessment at the 1-year follow-up except for age and McCabe score (Additional file 1: Table S3). Focal ARDS patients who completed the functional outcomes assessment were older (62 [14] vs. 54 [16] years; *p* = 0.005) and had a lower McCabe score (*p* = 0.027) than those who did not. However, there was no difference between focal and non-focal ARDS among patients who completed the functional outcomes assessment.

After a 1-year follow-up, the per-protocol cohort had significant impairment in patient-reported outcomes when compared against an age- and sex-matched population (Table 3) [24]. The median values for the standardised SF-36 physical and mental component summary were 37 (*p* < 0.001) and 41 (*p* < 0.001), respectively. Compared against an age- and sex-matched population [24], both focal (*p* < 0.001) and non-focal ARDS (*p* < 0.001) had impairment in the SF-36 physical component summary, with no differences between both groups (*p* = 0.201).

**Table 3:** One-year functional outcomes and quality of life, by subphenotype

Variables	Both groups* n = 147	Focal ARDS *n* = 60	Non-focal ARDS *n* = 87	*p* values^‡^
*Health-related quality of life: SF-36 (normalised score)*				
Physical functioning, median [IQR]	37 [21–47]	37 [21–47]	34 [21–48]	0.80
Physical component summary, median [IQR]	37 [31–46]	39 [34–45]	36 [30–47]	0.47
Mental health, median [IQR]	44 [38–52]	44 [35–52]	44 [38–51]	0.38
Mental component summary, median [IQR]	41 [34–49]	39 [32–48]	41 [36–51]	0.20
*Mental health symptoms: HAD*				
Anxiety subscale, median [IQR]	6 [3–10]	7 [4–10]	6 [3–9]	0.09
Patients with anxiety, n (%)	60 (41%)	27 (45%)	33 (38%)	0.44
Depression subscale, median [IQR]	6 [3–9]	6 [4–9]	6 [3–9]	0.23
Patients with depression, n (%)	51 (35%)	22 (37%)	29 (33%)	0.75
*Effect of fatigue on quality of life: MFIS*				
Physical functioning, median [IQR]	25 [18–30]	24 [15–29]	25 [18–30]	0.42
Cognitive functioning, median [IQR]	18 [10–25]	18 [10–26]	17 [10–25]	0.51
Psychosocial functioning, median [IQR]	5 [2–6]	5 [2–7]	5 [2–6]	0.68
MFIS component summary, median [IQR]	48 [33–59]	46 [35–61]	48 [32–58]	0.97
Patients with fatigue, *n* (%)	91 (62%)	34 (57%)	57 (66%)	0.43

Explanation of scoring: SF-36 normalised score (median = 50; SD = 10; range 0–100, higher score is better); HAD anxiety and depression subscale scores (range 0–21, lower score is better); presence of anxiety or depression was defined by a HAD subscale over 7; MFIS component summary (range 0–84, lower is better), MFIS subscale scores: physical (range 0–36), cognitive (range 0–40), psychosocial (range 0–8), fatigue was defined by a MFIS over 38

ARDS, acute respiratory distress syndrome; HAD, Hospital Anxiety and Depression Scale; IQR, interquartile range 25–75%; MFIS, Modified Fatigue Impact Scale; SF-36, Short Form 36 instrument

*Both groups reported patients in the per-protocol analysis

^‡^*p* values are reported between focal and non-focal ARDS (*p* value < 0.05 are in bold type)

Regarding the HAD scale, 41% and 35% of the per-protocol cohort had anxiety and depression, respectively. The median MFIS component summary of the per-protocol cohort was 43 (Table 3), and 91 (62%) patients experienced fatigue. There was no difference in anxiety and depression symptoms and rate of fatigue between the focal and non-focal ARDS groups (Table 3).

A sensitivity analysis dealing with patients that missed the 1-year functional assessment confirmed the previous analysis (Additional file 1: Table S4).

### Personalised ventilation protocol

At the 1-year follow-up, there was no difference in survival and quality of life between the personalised and the control groups regarding both the intention to treat and per-protocol analysis (Additional file 1: Fig. S2 and Tables S5 to S8).

## Discussion

To our knowledge, this is the first prospective study to assess the impact of morphological phenotypes on long-term outcomes among ARDS patients. We demonstrated a difference in survival over a 1-year follow-up between focal and non-focal ARDS, which is only driven by early mortality without any consequences of lung morphology beyond the first 90 days. Among survivors, physical and mental health, anxiety, depression, and fatigue were all impaired but independently of ARDS phenotypes.

### Impact of lung morphology phenotype on long-term outcomes

A prior study demonstrated a significant difference in early mortality between focal and non-focal ARDS without assessing long-term outcomes [8]. Beyond confirming these results, our study found that the difference in survival after a 1-year follow-up is only explained by short-term mortality. The impact of morphological phenotypes on mortality may be explained by differences in respiratory mechanics, with a higher driving pressure and elastance in non-focal ARDS [25]. Indeed, in our study, non-focal ARDS had higher plateau and driving pressures at baseline.

As with other intensive care variables (i.e. SAPS II, renal replacement therapy), which are independently associated with short-term mortality (90-day mortality), morphological phenotypes had no impact on mortality thereafter. Only comorbidities and previous health status seemed to impact long-term survival. These results are consistent with previous studies reporting that the severity of illness is a strong predictor of hospital and short-term mortality, whereas age, serious comorbidities, and previous health status are associated with long-term mortality in ARDS [11, 26]. Nevertheless, morphological phenotypes may be considered one of baseline intensive care variables, along with others such as respiratory mechanics and renal replacement therapy, which may only impact short-term mortality [27, 28]. These findings are consistent with a recent study that focused on the impact of the inflammatory phenotype on long-term survival in ARDS patients [12]. In that study, the inflammatory phenotype had an impact on short-term survival, with little implication after 90 days.

In our trial, beyond survival, quality of life was altered after a 1-year follow-up. Physical and mental health were impaired compared with a healthy population [24] which is consistent with previous studies [3, 21, 22]. Age and previous comorbidities have been reported to be associated with impairment of the SF-36 instrument [3]. Even though the specific contribution of ARDS is contested [29], the severity of the initial ARDS, the rapidity of its resolution, and the pulmonary dysfunction thereafter were all correlated with long-term outcomes [21, 30]. Anxiety and depression affected one-third of the ARDS survivors in our study. These results were similar to those of previous trials that reported between 11 and 42% of anxiety and 9 to 39% of depression using the HAD scale [22, 31]. Female sex, unemployment, alcohol misuse, and greater opioid use in the ICU were significantly associated with psychiatric symptoms [32]. Baseline intensive care variables, severity of illness, and mechanical ventilation duration were not associated with anxiety or depression. Eventually, fatigue was reported in 62% of ARDS survivors using the MFIS score in our study. Using another fatigue scale, a recent study reported 70% and 66% of ARDS survivors with significant symptoms of fatigue at 6-month and 1-year follow-ups, respectively [31]. Worse physical, cognitive, and mental health but not baseline critical care variables were associated with greater fatigue. Thus, one could wonder about the impact of morphological phenotypes on survivors’ later quality of life. However, in our study, there was no influence of morphological phenotypes on any functional score.

### Impact of a personalised ventilation protocol based on phenotypes on long-term outcomes

In the age of personalised medicine, a personalised ventilation protocol adjusted on the basis of morphological phenotypes was of concern. The LIVE trial failed to show any effect on 90-day mortality [10]. Similarly, we could not find any improvement in mortality or functional outcomes even with the exclusion of patients with a ventilation protocol break. The LIVE trial was unfortunately biased by a high number of misclassified patients who had worse outcomes than the control group. Morphological phenotyping of ARDS patients is a major problem because the misclassification of some patients makes it impossible to determine the value of phenotyping over the standard of care. Recently, some machine learning algorithms have been inconsistent in their ability to identify clusters of ARDS patients involved in significant heterogeneity of treatment effects [33]. Future studies may be needed to provide physicians with effective solutions to avoid misclassification, with or without the help of new technologies.

### Strengths and limits

The prospective and multicentre aspects of this study are some of its strengths. Compared with most studies, phenotyping was performed before randomisation, and the large cohort allowed the use of multivariable analysis in the statistical protocol, providing a more detailed interpretation of the data. However, this study has some limitations. First, it is a secondary analysis of a previous trial with an a priori sample size calculation based on the primary outcome and with numerous misclassified patients. This might have induced an underpower analysis, which can explain the absence of differences induced by the personalised ventilation protocol. Second, 24% of patients were misclassified into the focal and non-focal ARDS groups in the LIVE study, which may influence the different analyses and their interpretations. Indeed, misclassified patients have worse outcomes than correctly classified patients in both the LIVE trial and study [10]. Third, there were about 20% of missing data concerning plateau pressure, PEEP, and driving pressure. Given that we used a complete case analysis in our multivariable models, this may have induced a selection bias. However, our results were confirmed by a sensitive analysis with multiple imputations for missing data. Eventually, about 30% of survivors missed the functional outcome assessment after a 1-year follow-up. This may have induced a bias, especially as the patients were not similar in terms of age and McCabe score. However, our results are similar to those of previous studies, and there was no difference between the two phenotypes.

## Conclusion

Morphological phenotypes and 1-year mortality were associated with a higher mortality in patients with non-focal ARDS. This difference in survival was driven only by mortality before day 90. Our findings indicate that between day 90 and 1 year, none of the ICU parameters (severity scores, ventilator setting, lung morphology) had an impact on mortality and long-term outcomes. Yet, this study suggests that ARDS morphological phenotypes and related therapeutic approaches largely reflect the acute phase of the syndrome and its short-term impact on mortality; they do not impact the long-term outcomes of patients, which are only influenced by underlying comorbidities.

## Supplementary Information

**Additional file 1**. Method and Result supplements.

## Data Availability

Data collected for the study, including individual participant data, data dictionary defining each field in the set, and study protocol will be made available to others. Data will be communicated as de-identified participant data according to French law and will be available after publication of the manuscript. Data were stored at the Direction de la Recherche Clinique, University Hospital of Clermont-Ferrand, Clermont-Ferrand, France. Data requests should be addressed to Pr Jean-Michel Constantin (jean-michel.constantin@aphp.fr) who will send the data after receipt of a signed data access agreement.
