# Supplementary material for: One-year patient outcomes based on lung morphology in acute respiratory distress syndrome: secondary analysis of LIVE trial
Source: Crit Care. 2022 Jun 4;26:159. doi: 10.1186/s13054-022-04036-7 (PMC9166200; doi:10.1186/s13054-022-04036-7)
Supplement: Supplementary file 1 — Additional file 1. Method and Result supplements. [file 13054_2022_4036_MOESM1_ESM.docx]

**Additional File**

**To: One-year patient outcomes based on lung morphology in acute respiratory distress syndrome: secondary analysis of LIVE trial.**

**By Blanchard et al.**

**Content**

**Methods**

**Results**

- **Mortality and quality of life according to lung morphology**
  - **e-Table 1.** Cox-model result of the multivariable analysis for 1-year mortality regarding the impact of lung morphology phenotype.
  - **e-Table 2.** Cox-model result of the multivariate analysis for early and late mortality after multiple imputations for missing data.
  - **e-Table 3.** Demographic and clinical baseline characteristics of patients who survived after a 1-year follow-up with and without functional outcomes assessment in focal and non-focal ARDS.
  - **e-Table 4.** One-year functional outcomes and quality of life, by subphenotype after multiple imputations
- **Mortality and quality of life according to treatment group**
  - **e-Figure 1.** Flow chart of the intention-to-treat and per-protocol analysis
  - **e-Figure 2.** Effect of a personalised ventilator protocol on overall survival at 1 year.
  - **e-Table 5.** Cox-model result of the multivariable analysis for 1-year mortality in the intention-to-treat analysis regarding the impact of a ventilation protocol adjusted on the basis of lung morphology (personalised group) or to a standard strategy (control group).
  - **e-Table 6.** Cox-model result of the multivariable analysis for 1-year mortality in the per-protocol analysis regarding the impact of a ventilation protocol adjusted on the basis of lung morphology (personalised group) or to a standard strategy (control group).
  - **e-Table 7.** Demographic and clinical baseline characteristics of patients who survived after a 1-year follow-up with and without functional outcomes assessment in the personalised and control group
  - **e-Table 8.** 1-year functional outcomes and quality of life, by intervention group

**METHODS**

**Design and Patients**

In the LIVE trial, patients were randomly assigned to a ventilation protocol adjusted on the basis of lung morphology (personalised group) or to a standard strategy in line with traditional care (control group). The detailed protocol is available Supplemental data online. Patients assigned to the control group received, regardless of lung morphology, a tidal volume of 6 mL/kg of predicted body weight and a positive end expiratory pressure (PEEP) selected according to the low PEEP and FiO2 table from the ALVEOLI study [1]. For patients assigned to the personalised group, the ventilator settings were adjusted on the basis of lung morphology. Patients with focal ARDS received a tidal volume of 8 mL/kg predicted body weight with a PEEP of 5–10 cm of water, according to the oxygenation targets. Patients with non-focal ARDS received a tidal volume of 6 mL/kg predicted body weight, and PEEP was adjusted to reach a plateau pressure of 30 cm of water. Prone position was mandatory in the personalised group for patients with focal ARDS, was encouraged for all patients in the control group, and was usable only as rescue therapy in the personalised group for patients with non-focal ARDS. The detailed protocol of the LIVE trial is available elsewhere [2,3].

**Statistical analysis**

A sensitivity analysis with multiple imputations was conducted to deal with missing data. Missing data were analysed and considered missing completely at random. Multiple imputations for missing data were realized using the mice package. Two hundred imputed datasets were created using the predictive mean matching method. Distributions of original and imputed data were assessed using density plot. All variables were well matching with original data and distribution were considered to be similar. For multivariable analysis, a Cox model was applied to each of the imputed dataset. All results were thereafter pooled using the Rubin’s rules.

To assess the impact of a personalised ventilation protocol adjusted based on lung morphology (personalised group) compared to a standard strategy (control group) on mortality, the same variables used in the multivariable analysis of the LIVE study were included [2]. The Cox model included: morphological phenotype, age, haematological and non-haematological cancers comorbidities, Mac Cabe score, SAPS II without age (to avoid any interaction with age), PaO_2_/FiO_2_, mechanical ventilation duration (more or less than 48 hours), renal replacement therapy, shock, steroid use during the first 24 hours, intervention in the LIVE study, misclassification in morphological phenotype before LIVE study randomisation, and the centre of inclusion including a random effect to account for the centre effect. Because there was no missing date, the multivariable analysis was done on complete case.

We did both an intention-to-treat and a per-protocol analysis. The intention to-treat analysis included all participant who were randomly assigned to treatment (personalised ventilation protocol or standard strategy), except those who withdrew consent and those who were found to be ineligible because they met the exclusion. In the per-protocol analysis misclassified patients in the personalised group were excluded due to a breakdown in the ventilation protocol. Misclassified patients of the control group were not excluded because they were not misaligned with ventilator strategy, which, by definition, was not related to lung morphology.

A sensitivity analysis using imputation was realized to deal with missing data when comparing the functional outcomes. Imputations for missing data were realized using the mice package. Two hundred imputed datasets were created using the predictive mean matching method. Distributions of original and imputed data were assessed using density plot. All variables were well matching with original data and distribution were considered to be similar. Imputed data were added to the original dataset for descriptive analyse previously reported.

**RESULTS**

**Mortality and quality of life according to lung morphology**

**e-Table 1: Cox-model result of the multivariate analysis for 1-year mortality.**

| Predictors | Hazard ratio* | p value |
| --- | --- | --- |
| Non-Focal ARDS | 3.44 [1.80-6.59] | **< 0.001** |
| Age | 1.04 [1.02-1.06] | **< 0.001** |
| Male | 0.79 [0.46-1.34] | 0.377 |
| Haematological cancer | 2.24 [1.02-4.97] | **0.045** |
| Non-haematological cancer | 1.71 [0.88-3.34] | 0.113 |
| COPD | 1.51 [0.72-3.17] | 0.272 |
| Mac Cabe | 1.51 [1.04-2.19] | **0.029** |
| BMI | 0.98 [0.94-1.03] | 0.489 |
| SAPS II (without age) ^†^ | 1.02 [1.00-1.04] | **0.047** |
| PaO_2_/FiO_2_ | 1.00 [0.99-1.01] | 0.823 |
| Plateau pressure | 0.99 [0.77-1.28] | 0.968 |
| PEEP | 0.99 [0.77-1.28] | 0.966 |
| Driving pressure | 1.01 [0.79-1.31] | 0.906 |
| Shock | 1.29 [0.76-2.18] | 0.345 |
| Mechanical ventilation over 48h | 1.46 [0.59-3.57] | 0.413 |
| Steroid at admission | 1.27 [0.77-2.11] | 0.351 |
| Renal replacement therapy | 1.71 [1.04-2.79] | **0.033** |
| Intervention arm in LIVE study^$^ | 0.80 [0.51-1.27] | 0.349 |

p value < 0.05 are in bold type. *Results are reported as Hazard Ratio [CI95%]. ^†^SAPS II predictors include SAPS II without age due to the presence of the age already in the model. ^$^Intervention arm e explained in the LIVE study report^2^. ARDS: Acute respiratory distress syndrome, COPD: Chronic obstructive pulmonary disease, FiO_2_: Fraction of inspired Oxygen, CI95%: 95% confidence index, PaO_2_: Partial pressure of oxygen in arterial blood, PEEP: Positive end expiratory pressure, SAPS II: Simplified acute physiology score II.**e-Table 2: Cox-model result of the multivariate analysis for early and late mortality after multiple imputations for missing data.**

| Predictors | Early mortality | | Late mortality | |
| --- | --- | --- | --- | --- |
|  | **Hazard ratio*** | **p value** | **Hazard ratio*** | **p value** |
| Non-Focal ARDS | 2.00 [1.16-3.43] | **0.015** | 2.07 [0.74-5.84] | 0.230 |
| Age | 1.03 [1.01-1.05] | **0.001** | 1.03 [0.99-1.06] | 0.181 |
| Males | 1.02 [0.60-1.74] | 0.942 | 0.88 [0.31-2.47] | 0.817 |
| Haematological cancers | 1.69 [0.74-3.86] | 0.216 | 1.14 [0.12-10.76] | 0.914 |
| Non-haematological cancers | 1.84 [0.96-3.55] | 0.071 | 2.10 [0.71-6.15] | 0.239 |
| COPD | 1.19 [0.53-2.65] | 0.679 | 3.01 [0.93-9.73] | 0.128 |
| Mac Cabe | 1.22 [0.84-1.78] | 0.296 | 3.12 [1.49-6.50] | **0.032** |
| BMI | 1.00 [0.96-1.04] | 0.921 | 0.95 [0.87-1.05] | 0.360 |
| SAPS II (without age) † | 1.02 [1.01-1.04] | **0.012** | 1.01 [0.98-1.05] | 0.496 |
| PaO2/FiO2 | 1.00 [1.00-1.01] | 0.718 | 0.99 [0.98-1.00] | 0.148 |
| Plateau pressure | 1.07 [0.87-1.31] | 0.534 | 0.81 [0.58-1.13] | 0.274 |
| PEEP | 0.94 [0.76-1.15] | 0.532 | 1.15 [0.80-1.66] | 0.490 |
| Driving pressure | 0.95 [0.77-1.17] | 0.645 | 1.25 [0.88-1.77] | 0.272 |
| Shock | 1.10 [0.66-1.82] | 0.724 | 1.27 [0.50-3.22] | 0.634 |
| Mechanical ventilation over 48h | 2.83 [0.85-9.39] | 0.094 | 0.42 [0.13-1.40] | 0.221 |
| Steroid | 1.32 [0.81-2.15] | 0.277 | 1.54 [0.54-4.38] | 0.461 |
| Renal replacement therapy | 2.53 [1.58-4.05] | **<0.001** | 0.36 [0.08-1.65] | 0.248 |
| Intervention arm in LIVE study$ | 0.70 [0.44-1.12] | 0.141 | 2.00 [0.76-5.27] | 0.223 |

Early and late mortality were defined as mortality in the first 90 days and mortality between day 90 and one-year respectively (p value < 0.05 are in bold type). All patient in the per-protocol analysis were included (360 patients) including patients with missing data. Multiple imputations were made to deal with missing data. *Results are reported as Hazard Ratio [CI95%]. †SAPS II predictors include SAPS II without age due to the presence of the age already in the model. $Intervention arm is explained in the LIVE study report. ARDS: Acute respiratory distress syndrome, FiO2: Fraction of inspired Oxygen, CI95%: 95% confidence index, PaO2: Partial pressure of oxygen in arterial blood, SAPS II: Simplified acute physiology score II.

**e-Table 3: Demographic and clinical baseline characteristics of patients who survived after a 1-year follow-up with and without functional outcomes assessment in focal and non-focal ARDS.**

| Variables | Focal ARDS | | Non-Focal ARDS | |
| --- | --- | --- | --- | --- |
|  | **WITH functional assessment**  **n = 60** | **WITHOUT functional assessment**  **n = 27** | **WITH functional assessment**  **n = 87** | **WITHOUT functional assessment**  **n = 52** |
| ***BASELINE PATIENT DATAS*** | | | | |
| Age, years, mean [SD]^$^ | 62 [14] | 54 [16] | 60 [15] | 56 [16] |
| Male, n (%) | 46 (77%) | 23 (85%) | 66 (76%) | 33 (63%) |
| COPD, n (%) | 9 (15%) | 1 (4%) | 6 (7%) | 1 (2%) |
| Chronic renal failure, n (%) | 1 (2%) | 0 (0%) | 2 (2%) | 0 (0%) |
| Neoplasia, n (%) | 8 (13%) | 3 (11%) | 5 (6%) | 5 (10%) |
| Diabetes, n (%) | 6 (10%) | 3 (11%) | 7 (8%) | 7 (13%) |
| Arterial hypertension, n (%) | 12 (20%) | 1 (4%) | 20 (23%) | 14 (27%) |
| Smocking, n (%) | 1 (2%) | 0 (0%) | 3 (3%) | 2 (4%) |
| Alcohol disturbance, n (%) | 8 (13%) | 4 (15%) | 12 (14%) | 8 (15%) |
| Vasculopathy, n (%) | 12 (20%) | 2 (7%) | 18 (21%) | 8 (15%) |
| Cardiopathy, n (%) | 5 (8%) | 2 (7%) | 12 (14%) | 2 (4%) |
| BMI, kg/m^2^, mean [SD] | 28 [5] | 26 [5] | 26 [5] | 27 [6] |
| Mac Cabe score^$^ |  |  |  |  |
| 0, n (%) | 40 (67%) | 22 (81%) | 69 (79%) | 41 (79%) |
| 1, n (%) | 20 (33%) | 3 (11%) | 17 (20%) | 10 (19%) |
| 2, n (%) | 0 (0%) | 2 (7%) | 1 (1%) | 1 (2%) |
| ***BASELINE INTENSIVE CARE DATAS*** | | | | |
| Medical ICU, n (%) | 45 (75%) | 21 (78%) | 74 (85%) | 43 (83%) |
| SAPS II, mean [SD] | 51 [15] | 48 [18] | 48 [15] | 46 [16] |
| SOFA, mean [SD] | 10 [3] | 9 [4] | 8 [4] | 9 [3] |
| PaO_2_/FiO_2_, mean [SD] | 119 [38] | 110 [39] | 122 [44] | 113 [41] |
| Plateau pressure, cmH_2_O, mean [SD] | 24 [4] | 23 [5] | 24 [7] | 23 [5] |
| Driving pressure, cmH_2_O, mean [SD] | 13 [4] | 12 [5] | 14 [6] | 13 [5] |
| Shock at baseline, n (%)^‡^ | 42 (70%) | 15 (56%) | 41 (47%) | 33 (63%) |
| Primary lung injury^‡^ |  |  |  |  |
| Pneumonia, n (%) | 24 (40%) | 12 (44%) | 43 (49%) | 24 (46%) |
| Non-pulmonary infection, n (%) | 14 (23%) | 4 (15%) | 20 (23%) | 6 (12%) |
| Aspiration, n (%) | 15 (25%) | 9 (33%) | 15 (17%) | 19 (37%) |
| Trauma, n (%) | 1 (2%) | 2 (7%) | 3 (3%) | 0 (0%) |
| Other, n (%) | 6 (10%) | 0 (0%) | 6 (7%) | 3 (6%) |

Difference between patient with and without functional outcomes assessment at 1 year are reported as followed (those variables are referred as ^$^ in the table): Age (*Focal ARDS: p=0.007*; Non-Focal ARDS: p=0.093), Mac Cabe (*Focal ARDS: p=0.014*; Non-Focal ARDS: p=0.929). ^‡^Shock at baseline (Focal ARDS: p=0.190; Non-Focal ARDS: p=0.061), Primary lung injury (Focal ARDS: p=0.224; Non-Focal ARDS: p=0.057). ARDS: Acute respiratory distress syndrome, BMI: Body mass index, COPD: Chronic obstructive pulmonary disease, FiO_2_: Fraction of inspired Oxygen, ICU: Intensive Care Unit, LOS: Length of stay, PaO_2_: Partial pressure of oxygen in arterial blood, SAPS II: Simplified acute physiology score II, SD: Standard deviation, SOFA: Sequential Organ Failure Assessment score.

| Variables | Both Groups*  n = 226 | Focal ARDS  n = 87 | Non-Focal ARDS  n = 139 | p values^‡^ |  |
| --- | --- | --- | --- | --- | --- |
| **Health-related quality of life: SF-36 (normalised score)** | | | | | |
| Physical Functioning, median [IQR] | 35 [19-47] | 37 [19-47] | 34 [19-47] | 0.62 |  |
| Physical Component Summary, median [IQR] | 36 [30-46] | 38 [32-45] | 36 [30-46] | 0.52 |  |
| Mental Health, median [IQR] | 44 [36-52] | 44 [35-52] | 44 [37-51] | 0.46 |  |
| Mental Component Summary, median [IQR] | 41 [35-48] | 40 [34-47] | 41 [36-48] | 0.22 |  |
| **Mental health symptoms: HAD** | | | | | |
| Anxiety subscale, median [IQR] | 6 [4-9] | 7 [4-10] | 6 [3-9] | 0.09 |  |
| Patients with anxiety, n (%) | 96 (42%) | 40 (46%) | 56 (40%) | 0.40 |  |
| Depression subscale, median [IQR] | 6 [3-9] | 6 [4-9] | 5 [3-8] | 0.19 |  |
| Patients with depression, n (%) | 71 (31%) | 31 (36%) | 40 (29%) | 0.28 |  |
| **Effect of fatigue on quality of life: MFIS** | | | | | |
| Physical Functioning, median [IQR] | 25 [16-31] | 24 [15-30] | 25 [17-31] | 0.56 |  |
| Cognitive Functioning, median [IQR] | 18 [10-26] | 19 [10-26] | 17 [10-25] | 0.51 |  |
| Psychosocial Functioning, median [IQR] | 5 [2-6] | 5 [2-7] | 5 [2-6] | 0.41 |  |
| MFIS Component Summary, median [IQR] | 48 [35-59] | 47 [35-60] | 48 [34-59] | 0.66 |  |
| Patients with fatigue, n (%) | 149 (66%) | 56 (64%) | 93 (67%) | 0.70 |  |

**e-Table 4: One-year functional outcomes and quality of life, by subphenotype after multiple imputations**

All patient alive after a 1-year follow-up were included (226 patients) including patients with missing data (79 patients). Multiple imputations were made to deal with missing data. Explanation of scoring: SF-36 normalised score (median = 50; SD = 10; range: 0-100, higher score is better); HAD anxiety and depression subscale scores (range: 0-21, lower score is better), Presence of anxiety or depression were defined by a HAD subscale over 7; MFIS component summary (range: 0-84, lower is better), MFIS subscale scores: physical (range: 0-36), cognitive (range: 0-40), psychosocial (range: 0-8), fatigue was defined by a MFIS over 38. *Both groups reported patients in the per-protocol analysis. ^‡^p values are reported between Focal and Non-Focal ARDS (p value < 0.05 are in bold type). ARDS: Acute respiratory distress syndrome, HAD: Hospital Anxiety and Depression Scale, IQR: Interquartile range 25-75%, MFIS: Modified Fatigue Impact Scale, SF-36: Short Form 36 instrument.

**Mortality and quality of life according to treatment group**

**Population**

From June 2014 to February 2017, 420 patients were included in the LIVE study. Twenty patients were excluded as described in the e-Figure 1. Of the remaining 400 patients, 204 were in the control group and 196 in the personalised group. Forty patients were misclassified in the personalised group and were excluded from the per-protocol analysis. Demographic characteristics and ICU baseline data are available in a previous report^1^

**Mortality according to treatment group**

In the intention-to-treat analysis there were no difference of mortality between the personalised and the control group (1-year mortality: 68 (38%) versus 66 (34%), log-rank test: p=0.633) (e-Figure 2A). In the multivariable analysis, non-focal ARDS (Hazard ratio (HR), 2.11; 95% confidence interval (95%CI), 1.39-3.20; p<0.001), Age, (HR, 1.02; (95%CI), 1.01-1.04; p=0.003), Mac Cabe score (HR, 1.53; (95%CI), 1.13-2.07; p=0.006), SAPS II without age (HR, 1.02; (95%CI), 1.00-1.03; p=0.018), a renal replacement therapy (HR, 2.35; (95%CI), 1.60-3.44; p<0.001), and a misclassification in morphological phenotype before randomisation in the LIVE study (HR, 2.74; (95%CI), 1.75-4.27; p<0.001) were independently associated with 1-year mortality (e-Table 3). The personalised group, haematological and non-haematological cancer, PaO_2_/FiO_2_, mechanical ventilation over 48h, steroid use at admission were not (e- Table 5).

In the per-protocol analysis (after excluding 40 misclassified patients in the personalised group), there were no difference of mortality between the personalised and the control group (1-year mortality: 42 (30%) versus 66 (34%), log-rank test: p=0.212) (e-Figure 2B). In the multivariable analysis, non-focal ARDS (HR, 1.78; (95%CI), 1.14-2.79; p=0.012), Age (HR, 1.02; (95%CI), 1.01-1.04; p=0.005), Mac Cabe score (HR, 1.58; (95%CI), 1.14-2.20; p=0.007), SAPS II (HR, 1.02; (95%CI), 1.00-1.03, p=0.025), a renal replacement therapy (HR, 2.02; (95%CI), 1.32-3.09; p=0.001) were independently associated with 1-year mortality (e-Table 4). The personalised group, haematological and non-haematological cancer, PaO_2_/FiO_2_, mechanical ventilation over 48h, steroid use at admission, and a misclassification in morphological phenotype before randomisation in the LIVE study were not (e-Table 6).

**Misclassified patients**

In the personalised group, mortality was higher in case of misclassification for both focal (log-rank test: p < 0.001) and non-focal ARDS (log-rank test: p=0.001) (e- Figure 2D). In the control group, mortality wasn’t different in case of misclassification for both focal (log-rank test: p=0.648) and non-focal ARDS (log-rank test: p=0.603) (e-Figure 2C). Misclassification was independently associated with 1-year mortality in the intention-to-treat analysis (HR, 2.74; (95%CI), 1.75-4.27; p<0.001) but not in the per-protocol analysis (HR, 1.55; (95%CI), 0.86-2.82; p=0.147) which may be interpreted as an association between mortality and misclassification in the personalised group.

**Quality of life after according to treatment group**

Over the 400 patients in the intention-to-treat analysis, 238 patients survived after a 1-year follow-up and were eligible for functional outcomes assessment. Eighty-four patients (35%) missed the functional outcomes assessment (50 [39%] and 34 [31%] patients in the control and personalised group respectively). There was no difference between patients who did and did not complete the functional outcomes assessment at 1 year except for age and history of COPD and the primary lung injury (e- Table 7). Patient in the personalised group who did complete the functional outcomes assessment were older (62 [14] versus 57 [15] years, p=0.043) than those would did not. Patient in the control group who did complete the functional outcomes assessment had more COPD (7 [9%] versus 0 [0%], p=0.028) and had different causes of primary lung injury (p=0.042) than those would did not. However, within patients that complete the functional outcomes assessment, there was no difference between patients in the personalised and the control group.

After a 1-year follow-up, the intention to treat cohort had significant impairment in patients-reported outcomes when compared against an age- and sex-matched population [4] (e-Table 8). Indeed, the median value for the standardised (mean=50, standard deviation=10) SF36 physical and mental component summary were 37 (p<0.001) and 44 (p<0.001), respectively. Compared against an age- and sex-matched population [4], both the personalised (p<0.001) and control group (p<0.001) had impairment in the SF36 physical component summary without difference between both groups (p=0.681).

Using the HAD scale, the intention to treat cohort had anxiety and depression in 41% and 35%, respectively. There was no difference between both groups for anxiety and depression symptoms (e-Table 8). Median MFIS Component Summary was 48 in the intention to treat cohort (e-Table 8). There was no difference between focal and non-focal ARDS patients for the MFIS Component Summary and all its subclass. Ninety-five patients (62%) had fatigue in the entire cohort without any difference between both groups.

Same results were found using the per-protocol analysis (excluding 40 misclassified patients in the personalised group) and are not reported.

**
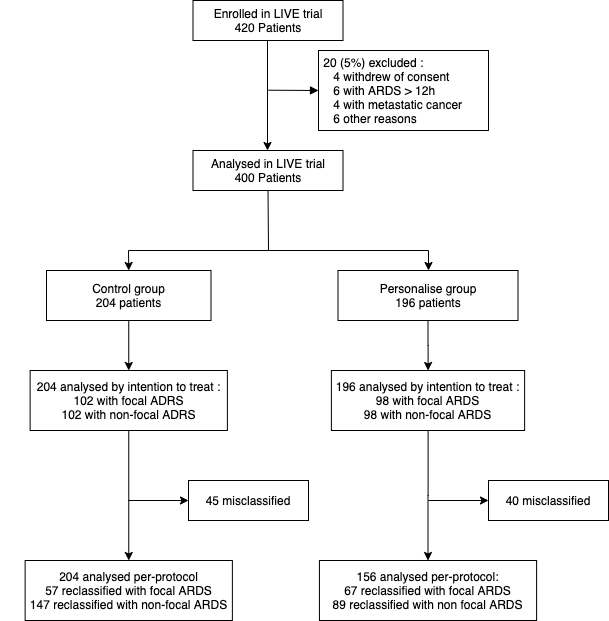
**

**e-Figure 1: Flow chart:** Shown is the recruitment of the cohort for in the intention-to-treat and per-protocol analysis. The intention to-treat analysis included all participant who were randomly assigned to treatment (personalised ventilation protocol or standard strategy), except those who withdrew consent and those who were found to be ineligible because they met the exclusion. In the per-protocol analysis misclassified patients in the personalised group were excluded due to a breakdown in the ventilation protocol. Misclassified patients of the control group were not excluded because they were not misaligned with ventilator strategy, which, by definition, was not related to lung morphology.

***
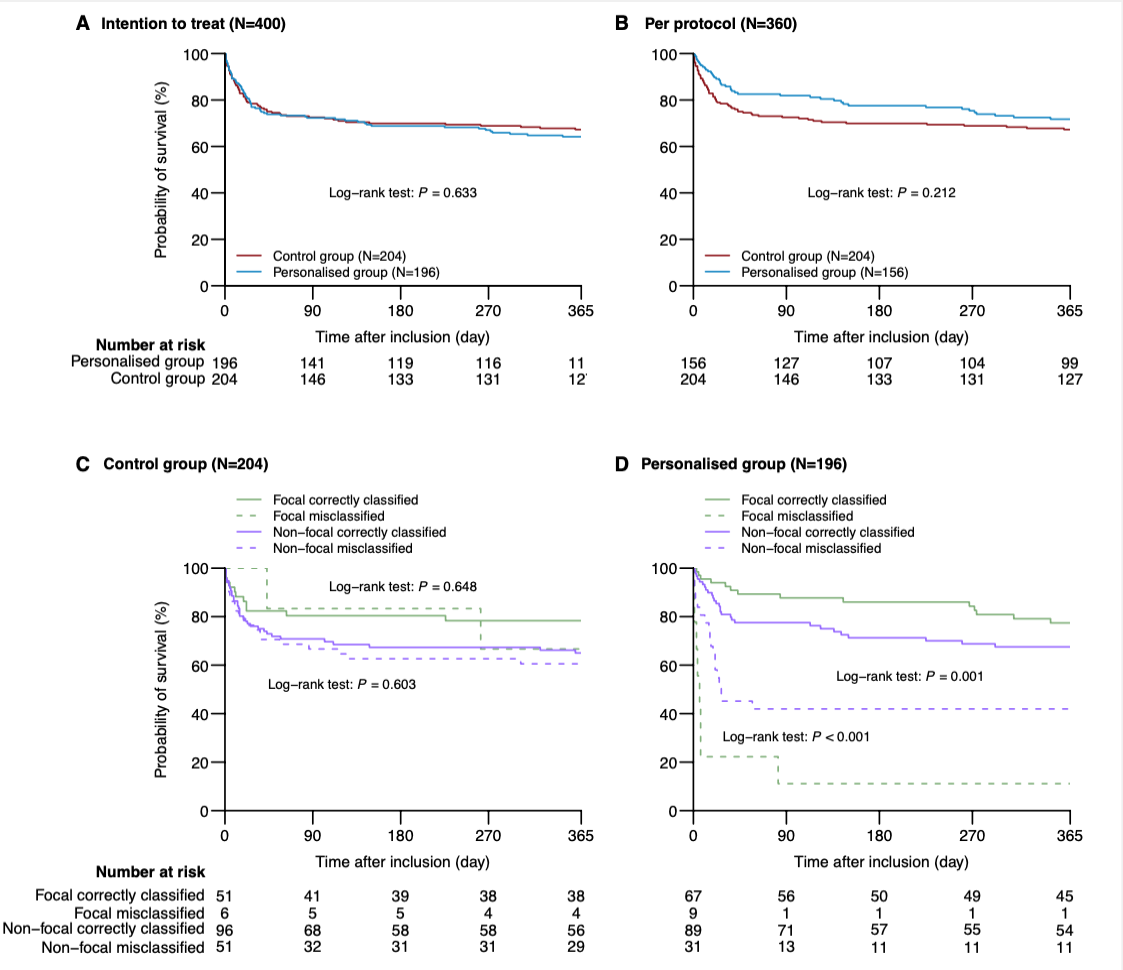
***

**e-Figure 2: Effect of a personalised ventilator protocol on overall survival at 1 year. A.** Survival as established by the intention-to-treat analysis, considering all the patients, regardless of whether they were correctly classified or misclassified. **B.** Survival as established by the per-protocol analysis, only considering patients who were correctly classified in the personalised group. **C.** Survival for patients with focal and non-focal ARDS who were correctly classified or misclassified in the control group. There was no difference in mortality between ARDS who were correctly classified or misclassified. **D.** Survival for patients with focal and non-focal ARDS who were correctly classified or misclassified in the personalised group. Mortality in misclassified focal and non-focal ARDS was higher than in correctly classified ones (Focal ARDS: log-rank test: p<0.001, Non-focal ARDS: log-rank test: p=0.001). Survival curves were realised using the Kaplan-Meyer methods. ARDS: acute respiratory distress syndrome.

**e-Table 5. Cox-model result of the multivariable analysis for 1-year mortality in the intention-to-treat analysis regarding the impact of a ventilation protocol adjusted on the basis of lung morphology (personalised group) or to a standard strategy (control group).**

| Predictors | Hazard ratio* | p value |
| --- | --- | --- |
| Personalised group^$^ | 1.47 [0.93-1.89] | 0.115 |
| Non-Focal ARDS | 2.11 [1.39-3.20] | **< 0.001** |
| Age | 1.02 [1.01-1.04] | **0.003** |
| Haematological cancers | 1.38 [0.67-2.80] | 0.380 |
| Non-haematological cancers | 1.69 [1.00-2.85] | 0.051 |
| Mac Cabe | 1.53 [1.13-2.07] | **0.006** |
| SAPS II without age^†^ | 1.02 [1.00-1.03] | **0.018** |
| PaO2/FiO2 | 1.00 [0.99-1.00] | 0.280 |
| Shock | 1.26 [0.83-1.91] | 0.281 |
| Mechanical ventilation over 48h | 1.50 [0.69-3.25] | 0.305 |
| Renal replacement therapy | 2.35 [1.60-3.44] | **< 0.001** |
| Steroid at admission | 1.38 [0.93-2.06] | 0.109 |
| Misclassification before randomisation^$^ | 2.74 [1.75-4.27] | **< 0.001** |

P value < 0.05 are in bold type. *Results are reported as Hazard Ratio [CI95%]. ^†^SAPS II predictors include SAPS II without age due to the presence of the age already in the model. ^$^Intervention arm and misclassification are explained in the LIVE study report [2]. ARDS: Acute respiratory distress syndrome, FiO_2_: Fraction of inspired Oxygen, CI95%: 95% confidence index, PaO_2_: Partial pressure of oxygen in arterial blood, SAPS II: Simplified acute physiology score II.

**e-Table 6. Cox-model result of the multivariable analysis for 1-year mortality in the per-protocol analysis regarding the impact of a ventilation protocol adjusted on the basis of lung morphology (personalised group) or to a standard strategy (control group).**

| Predictors | Hazard ratio* | p value |
| --- | --- | --- |
| Personalised group^$^ | 0.92 [0.59-1.42] | 0.697 |
| Non-Focal ARDS | 1.78 [1.14-2.79] | **0.012** |
| Age | 1.02 [1.01-1.04] | **0.005** |
| Haematological cancers | 1.45 [0.65-3.22] | 0.366 |
| Non-haematological cancers | 1.68 [0.95-2.96] | 0.072 |
| Mac Cabe | 1.58 [1.14-2.20] | **0.007** |
| SAPS II without age^†^ | 1.02 [1.00-1.03] | **0.025** |
| PaO2/FiO2 | 1.00 [0.99-1.00] | 0.833 |
| Shock | 1.23 [0.79-1.92] | 0.367 |
| Mechanical ventilation over 48h | 1.55 [0.70-3.43] | 0.276 |
| Renal replacement therapy | 2.02 [1.32-3.09] | **0.001** |
| Steroid at admission | 1.38 [0.88-2.16] | 0.165 |
| Misclassification before randomisation^$^ | 1.55 [0.86-2.82] | 0.147 |

P value < 0.05 are in bold type. *Results are reported as Hazard Ratio [CI95%]. ^†^SAPS II predictors include SAPS II without age due to the presence of the age already in the model. ^$^Intervention arm and misclassification are explained in the LIVE study report [2]. ARDS: Acute respiratory distress syndrome, FiO_2_: Fraction of inspired Oxygen, CI95%: 95% confidence index, PaO_2_: Partial pressure of oxygen in arterial blood, SAPS II: Simplified acute physiology score II.

**e-Table 7: Demographic and clinical baseline characteristics of patients who survived after a 1-year follow-up with and without functional outcomes assessment in the personalised and control group.**

| Variables | Control group | | Personalised group | |
| --- | --- | --- | --- | --- |
|  | **WITH functional assessment**  **n = 77** | **WITHOUT functional assessment**  **n = 50** | **WITH functional assessment**  **n = 77** | **WITHOUT functional assessment**  **n = 34** |
| ***BASELINE PATIENT DATAS*** | | | | |
| Age, years, mean [SD]^$^ | 60 [15] | 55 [17] | 62 [14] | 57 [15] |
| Males, n (%) | 56 (73%) | 36 (72%) | 62 (81%) | 24 (71%) |
| COPD, n (%)^$^ | 7 (9%) | 0 (0%) | 10 (13%) | 3 (9%) |
| Chronic renal failure, n (%) | 0 (0%) | 3 (6%) | 3 (4%) | 0 (0%) |
| Neoplasia, n (%) | 6 (8%) | 6 (12%) | 8 (10%) | 2 (6%) |
| Diabetes, n (%) | 5 (6%) | 5 (10%) | 9 (12%) | 5 (15%) |
| Arterial hypertension, n (%) | 14 (18%) | 7 (14%) | 19 (25%) | 8 (24%) |
| Smocking, n (%) | 3 (4%) | 1 (2%) | 1 (1%) | 1 (3%) |
| Alcohol disturbance, n (%) | 13 (17%) | 6 (12%) | 9 (12%) | 6 (18%) |
| Vasculopathy, n (%) | 14 (18%) | 7 (14%) | 18 (23%) | 5 (15%) |
| Cardiopathy, n (%) | 8 (10%) | 2 (4%) | 10 (13%) | 4 (12%) |
| BMI, kg/m^2^, mean [SD] | 27 [5] | 26 [5] | 27 [5] | 27 [5] |
| Mac Cabe score |  |  |  |  |
| 0, n (%) | 61 (79%) | 38 (76%) | 54 (70%) | 28 (82%) |
| 1, n (%) | 15 (19%) | 9 (18%) | 23 (30%) | 6 (18%) |
| 2, n (%) | 1 (1%) | 3 (6%) | 0 (0%) | 0 (0%) |
| ***BASELINE INTENSIVE CARE DATAS*** | | | | |
| Medical ICU, n (%) | 61 (79%) | 41 (82%) | 62 (81%) | 27 (79%) |
| SAPS II, mean [SD] | 50 [15] | 45 [16] | 48 [15] | 50 [16] |
| SOFA, mean [SD] | 9 [3] | 8 [3] | 9 [4] | 9 [4] |
| PaO_2_/FiO_2_, mean [SD] | 115 [42] | 115 [37] | 125 [40] | 111 [44] |
| Plateau pressure, cmH_2_O, mean [SD] | 23 [6] | 23 [5] | 25 [5] | 23 [5] |
| Driving pressure, cmH_2_O, mean [SD] | 13 [4] | 13 [5] | 14 [6] | 13 [4] |
| Shock at baseline, n (%) | 44 (57%) | 28 (56%) | 42 (55%) | 21 (62%) |
| Primary lung injury^$^ |  |  |  |  |
| Pneumonia, n (%) | 33 (43%) | 24 (48%) | 38 (49%) | 15 (44%) |
| Non-pulmonary infection, n (%) | 21 (27%) | 6 (12%) | 14 (18%) | 4 (12%) |
| Aspiration, n (%) | 12 (16%) | 17 (34%) | 19 (25%) | 12 (35%) |
| Trauma, n (%) | 3 (4%) | 1 (2%) | 1 (1%) | 1 (3%) |
| Other, n (%) | 8 (10%) | 2 (4%) | 5 (6%) | 2 (6%) |

Difference between patient with and without functional outcomes assessment at 1 year are reported as followed (those variable are referred as ^$^ in the table): Age (Control group: p=0.130; *Personalised group: p=0.043*), COPD (*Control group: p=0.028*; Personalised group: p=0.530), Primary lung injury (*Control group: p=0.042*; Personalised group: p=0.726*)*. ARDS: Acute respiratory distress syndrome, BMI: Body mass index, COPD: Chronic obstructive pulmonary disease, FiO_2_: Fraction of inspired Oxygen, ICU: Intensive Care Unit, LOS: Length of stay, PaO_2_: Partial pressure of oxygen in arterial blood, SAPS II: Simplified acute physiology score II, SD: Standard deviation, SOFA: Sequential Organ Failure Assessment score.

| Variables | Both Groups*  n = 154 | Control group  n = 77 | Personalised group  n = 77 | p values^‡^ |  |
| --- | --- | --- | --- | --- | --- |
| **Health-related quality of life: SF-36 (normalised score)** | | | | | |
| Physical Functioning, median [IQR] | 37 [21-47] | 37 [24-47] | 34 [19-47] | 0.53 |  |
| Physical Component Summary, median [IQR] | 37 [31-46] | 37 [31-46] | 37 [30-46] | 0.46 |  |
| Mental Health, median [IQR] | 44 [36-52] | 44 [38-50] | 46 [36-54] | 0.35 |  |
| Mental Component Summary, median [IQR] | 41 [34-49] | 39 [34-47] | 43 [34-51] | 0.51 |  |
| **Mental health symptoms: HAD** | | | | | |
| Anxiety subscale, median [IQR] | 6 [3-10] | 6 [4-9] | 6 [3-11] | 0.38 |  |
| Patients with anxiety, n (%) | 63 (41%) | 32 (42%) | 31 (40%) | 0.95 |  |
| Depression subscale, median [IQR] | 6 [3-9] | 6 [4-9] | 6 [3-9] | 0.31 |  |
| Patients with depression, n (%) | 54 (35%) | 26 (34%) | 28 (36%) | 0.91 |  |
| **Effect of fatigue on quality of life: MFIS** | | | | | |
| Physical Functioning, median [IQR] | 25 [17-30] | 24 [18-30] | 25 [16-30] | 0.68 |  |
| Cognitive Functioning, median [IQR] | 18 [10-25] | 18 [10-24] | 18 [9-26] | 0.87 |  |
| Psychosocial Functioning, median [IQR] | 5 [2-6] | 5 [2-6] | 5 [2-6] | 0.85 |  |
| MFIS Component Summary, median [IQR] | 48 [33-59] | 48 [34-58] | 47 [31-60] | 0.34 |  |
| Patients with fatigue, n (%) | 95 (62%) | 49 (64%) | 46 (60%) | 0.59 |  |

**e-Table 8: 1-year functional outcomes and quality of life, by intervention group**

Explanation of scoring: SF-36 normalised score (median = 50; SD = 10; range: 0-100, higher score is better); HAD anxiety and depression subscale scores (range: 0-21, lower score is better), Presence of anxiety or depression were defined by a HAD subscale over 7; MFIS component summary (range: 0-84, lower is better), MFIS subscale scores: physical (range: 0-36), cognitive (range: 0-40), psychosocial (range: 0-8), fatigue was defined by a MFIS over 38. *Both groups reported patients in the per-protocol analysis. ^‡^p values are reported between Focal and Non-Focal ARDS. ARDS: Acute respiratory distress syndrome, HAD: Hospital Anxiety and Depression Scale, IQR: Interquartile range 25-75%, MFIS: Modified Fatigue Impact Scale, SF-36: Short Form 36 instrument**.**

**REFERENCES**

1. Heart TN. Higher versus Lower Positive End-Expiratory Pressures in Patients with the Acute Respiratory Distress Syndrome. New Engl J Medicine. 2004;351:327–36.

2. Constantin J-M, Jabaudon M, Lefrant J-Y, Jaber S, Quenot J-P, Langeron O, et al. Personalised mechanical ventilation tailored to lung morphology versus low positive end-expiratory pressure for patients with acute respiratory distress syndrome in France (the LIVE study): a multicentre, single-blind, randomised controlled trial. Lancet Respir Medicine. 2019;7:870–80.

3. Jabaudon M, Godet T, Futier E, Bazin J-É, Sapin V, Roszyk L, et al. Rationale, study design and analysis plan of the lung imaging morphology for ventilator settings in acute respiratory distress syndrome study (LIVE study): Study protocol for a randomised controlled trial. Anaesth Crit Care Pa. 2017;36:301–6.

4. Leplège A, Ecosse E, Verdier A, Perneger TV. The French SF-36 Health Survey Translation, Cultural Adaptation and Preliminary Psychometric Evaluation. J Clin Epidemiol. 1998;51:1013–23.
